# Cytokeratin-19 positivity is acquired along cancer progression and does not predict cell origin in rat hepatocarcinogenesis

**DOI:** 10.18632/oncotarget.5501

**Published:** 2015-10-03

**Authors:** Marta Anna Kowalik, Pia Sulas, Giovanna Maria Ledda-Columbano, Silvia Giordano, Amedeo Columbano, Andrea Perra

**Affiliations:** ^1^ Department of Biomedical Sciences, University of Cagliari, Cagliari, Italy; ^2^ University of Torino School of Medicine, Candiolo Cancer Institute-FPO, IRCCS Candiolo, Torino, Italy

**Keywords:** preneoplastic foci, oval cells, Nrf2/Keap1, hepatocarcinogenesis, cell origin

## Abstract

Although the expression of the stem/progenitor cell marker cytokeratin-19 (CK-19) has been associated with the worst clinical prognosis among all HCC subclasses, it is yet unknown whether its presence in HCC is the result of clonal expansion of hepatic progenitor cells (HPCs) or of de-differentiation of mature hepatocytes towards a progenitor-like cell phenotype. We addressed this question by using two rat models of hepatocarcinogenesis: the Resistant-Hepatocyte (R-H) and the Choline-methionine deficient (CMD) models. Our data indicate that the expression of CK-19 is not the result of a clonal expansion of HPCs (oval cells in rodents), but rather of a further step of preneoplastic hepatocytes towards a less differentiated phenotype and a more aggressive behavior. Indeed, although HCCs were positive for CK-19, very early preneoplastic foci (EPFs) were completely negative for this marker. While a few weeks later the vast majority of preneoplastic nodules remained CK-19 negative, a minority became positive, suggesting that CK-19 expression is the result of de-differentiation of a subset of EPFs, rather than a marker of stem/progenitor cells. Moreover, the gene expression profile of CK-19-negative EPFs clustered together with CK-19-positive nodules, but was clearly distinct from CK-19 negative nodules and oval cells.

**Conclusion:**

i) CK-19-positive cells are not involved in the early clonal expansion observed in rat hepatocarcinogenesis; ii) CK-19 expression arises in preneoplastic hepatocyte lesions undergoing malignant transformation; iii) CK-19 positivity in HCCs does not necessarily reflect the cell of origin of the tumor, but rather the plasticity of preneoplastic cells during the tumorigenic process.

## INTRODUCTION

The heterogeneous nature of human hepatocellular carcinoma (HCC), which represents a serious social health problem [1], has so far impeded both treatment strategies and prognostic predictions [2]. Even though HCC is considered to emerge through a process of multistep carcinogenesis [3], its histogenesis remains a subject of discussion and controversy. In fact, other than the different etiologic factors, an important cause of liver cancer heterogeneity may be the cell type of origin. Besides a long-established idea that preneoplastic lesions arise exclusively from mature hepatocytes undergoing neoplastic transformation [4–7], it has been also hypothesized that a subset of HCC can originate from hepatic progenitor cells (HPC) [8, 9]. This subclass of human HCC results enriched for genes expressed in fetal hepatoblasts, including some progenitor cell markers. A progressive up-regulation of HPCs was also demonstrated in dysplastic nodules in human liver [10] and hepatocellular adenoma [11]. Moreover, numerous studies have reported HPCs activation in the most relevant liver carcinogenic conditions in the Western world, such as chronic viral hepatitis and alcoholic and non-alcoholic fatty liver disease [12].

Although at present no single antibody can unambiguously define hepatic progenitor cells, several markers have been proposed for HPCs in HCC. In contrast to hepatocytes, HPCs are thought to express biliary markers, such as cytokeratin-7 (CK-7), cytokeratin-19 (CK-19), and Ov6; moreover, a subset of these cells expresses fetal hepatocyte (α-fetoprotein) and hematopoietic markers (c-kit, CD34) [8, 13].

Over the past few years, cytoskeleton-associated proteins have been well recognized as cellular integrators in the neoplastic process [14]. Different epithelia express characteristic combinations of cytokeratin proteins, depending on the organ of origin or the type of differentiation [15]. For example, in normal liver, hepatocytes express CK-8 and CK-18, whereas biliary epithelial cells express CK-7 and CK-19 [16, 17] as well. Recently, ‘stemness’-related markers have also become of particular relevance, as they can successfully predict the clinical outcome of HCC patients. In particular, the CK-19-positive HCC subtype is characterized by the worst clinical prognosis among all HCC subclasses, suggesting that CK-19 is a negative prognostic marker for HCC [9, 18]. CK-19 expression was also correlated with some clinicopathological features, such as poor tumor differentiation, metastasis, early tumor recurrence after resection and radiofrequency ablation [19–22]. Furthermore, CK-19-positive HCCs show significantly increased epithelial-mesenchymal transition (EMT) and expression of invasion-related molecules, suggesting that they are endowed with more invasive characteristics, compared to CK-19-negative HCCs [23]. In a recent study, Govaere *et al.* [24] demonstrated that *in vitro* primary human CK-19-positive tumor cells showed increased invasiveness and that CK-19 knockdown significantly reduced HCC invasive ability and rendered HCC cells more sensitive to cytotoxic agents, such as doxorubicin, 5-fluorouracil and sorafenib.

An important support to the HPC hypothesis was derived mainly from rodent models of chemical hepatocarcinogenesis [25, 26]. In these models, a peri-portal population of small epithelial cells, called oval cells, related to terminal biliary ductules and canals of Hering was described. This cell population has the ability to differentiate towards hepatocytes, bile ductular cells and intestinal epithelium and can give rise to hepatocellular carcinoma and cholangiocellular carcinoma.

A well-characterized rat hepatocarcinogenesis model is the Resistant-Hepatocyte (R-H) model, in which tumors are initiated by a single dose of a chemical carcinogen (diethylnitrosamine, DENA) and promoted by a brief treatment with 2-acetylaminofluorene (2-AAF) combined with partial hepatectomy (PH) [27]. The R-H model offers the possibility to identify distinct lesions (preneoplastic foci, preneoplastic nodules, early and fully developed HCCs, and occasional features of combined hepato-cholangiocarcinomas) at well-defined timings. Interestingly, a modification of the R-H model, consisting in the omission of DENA initiation, has been extensively used in studies of activation, expansion and differentiation of oval cells [28]. Therefore, the advantage of the R-H model is that it allows not only examining the expansion of both preneoplastic and oval cells at the same time, but also investigating the evolution of the early preneoplastic lesions to fully developed HCC. Recently, comparative functional genomics has shown a stringent clustering of CK-19-positive preneoplastic nodules and advanced HCCs obtained from the R-H model with human HCCs characterized by poor prognosis [29, 30].

Although these findings suggest that CK-19-positive HCCs could originate from progenitor cells, some reports cast doubt on the progenitor cell origin of CK-19-positive HCC [31, 32]. Therefore, it remains elusive whether the expression of CK-19, as well as of other HPC markers, represents i) retention of a progenitor cell phenotype all throughout the carcinogenic process or ii) the result of de-differentiation of preneoplastic or malignant hepatocytes to a progenitor cell/biliary phenotype during progression towards HCC.

The aim of the present study was to analyze the early changes in the R-H model of carcinogenesis in order to investigate the relationship between oval cell proliferation and EPFs, as well as to understand whether in this protocol CK-19 expression is built-in in clonally expanding EPFs or if it is acquired through a progressive de-differentiation of preneoplastic hepatocytes towards a progenitor cell phenotype. The latter question was also addressed by using another rat model of hepatocarcinogenesis consisting of a chronic exposure to a steatogenic environment generated by a choline devoid-methionine deficient diet (CMD) [33].

## RESULTS

### mRNA expression profiling of oval cells is distinct from that of early preneoplastic foci (EPFs)

**Figure 1:**
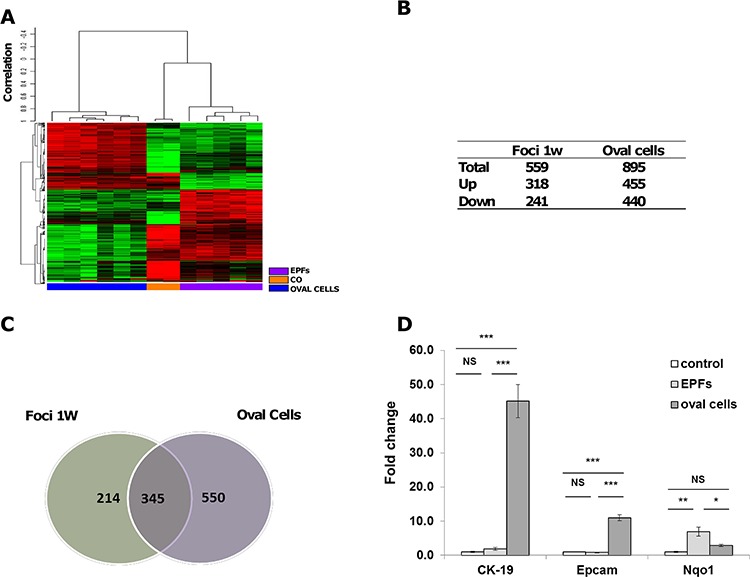
mRNA profile of oval cells and EPFs in the R-H model of hepatocarcinogenesis **A.** Hierarchical clustering of 1,570 genes in normal liver (CO), laser-microdissected oval cells and early preneoplastic foci (EPFs) 7 days after PH. Each row represents the expression profile of a gene and each column represents a sample from pools of microdissected oval cells or EPFs. Controls and different types of lesions are indicated by colored bars. Only mRNAs whose expression was dysregulated more than 2-fold were considered. Red and green colors represent higher or lower expression levels of the mRNA (median-centered), respectively. **B.** Number of differentially expressed mRNAs in oval cells and EPFs compared to age-matched controls, defined by *P* < 0.05 (BH-corrected). RNA was extracted from laser-microdissected oval cells and EPFs occurring 7 days after PH and CK-19-positive and CK-19-negative nodules generated 7 weeks after surgery. **C.** Venn diagrams illustrating significantly dysregulated genes in oval cells and EPFs. **D.** QRT-PCR validation of CK-19, Epcam and Nqo1 in controls, oval cells and EPFs. Gene expression is reported as fold-change relative to age-matched controls. ***P < 0.001, **P < 0.01, *P < 0.05, NS: not significant.

While several works suggest that oval cells can differentiate to hepatocytes [25, 26, 28], the role of oval cells in the development of HCC is a controversial matter [4, 5, 31, 34–37]. To explore this issue, we used the Resistant-Hepatocyte (R-H) rat model [27] (Supp. Figure 1A). This model is characterized by a synchronous expansion of carcinogen-initiated cells that can be easily identified by preneoplastic markers (such as the expression of GSTP) as early as 3–7 days after PH. Unlike normal hepatocytes, preneoplastic hepatocytes are able to divide after 2/3 PH, in the presence of the cytostatic environment generated by 2-AAF. Concomitantly, resection of 2/3 of the liver in the presence of 2-AAF causes a massive expansion of oval cells, expressing markers specific for biliary epithelial cells, such as CK-19, GSTP and gamma-glutamyltranspeptidases. Notably, while oval cells, like GSTP-positive hepatocyte preneoplastic lesions, are completely absent prior to PH, a concomitant surge of both these two cell populations is observed 2–3 days after PH. Using this model, we performed gene expression profiling in micro-dissected oval cells and early preneoplastic foci (EPFs) appearing 7 days after PH. EPFs appeared as small spherical lesions consisting of 15 to 100 hepatocytes, with basophilic cells characterized by prominent nucleoli. Mitotic figures were often present. A total of 1,570 out of 21,791 genes included in the array were detected, according to the parameters described in Supplementary Material. Hierarchical cluster analysis stratified rat lesions into two major clusters: 1) oval cells; 2) normal liver and EPFs; within the second cluster, normal liver (CO) and EPFs formed 2 distinct sub-clusters (Figure 1A). Notably, among the significantly dysregulated genes (at least 2 fold change difference), 69% were exclusive of either oval cells or EPFs (Figure 1B, 1C). Quantitative RT-PCR validation performed on a few genes typically expressed by oval cells (Epcam and CK-19) or mainly expressed by mature hepatocytes (Nqo1) confirmed microarray expression data (Figure 1D). These results show that oval cells have an expression profile clearly distinct from that of hepatocyte EPFs and suggest that they are not the cells of origin of EPFs.

### Oval cells but not early preneoplastic foci (EPF) show positivity to CK-19

**Figure 2:**
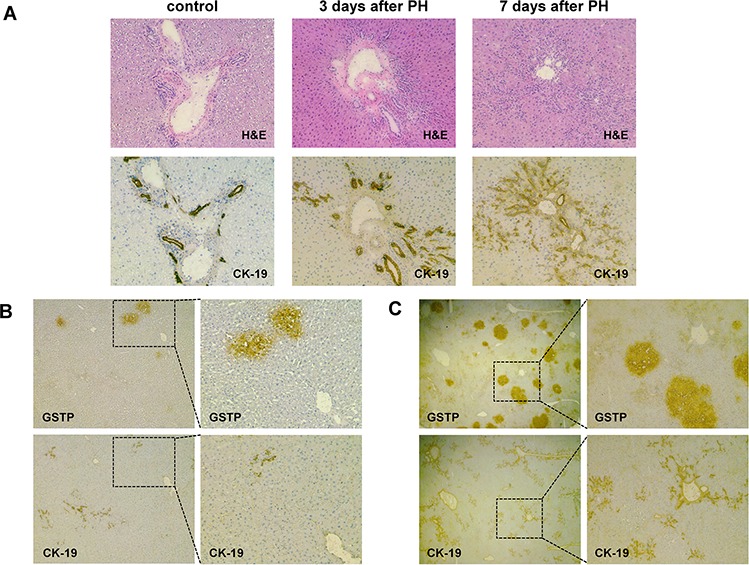
Early preneoplastic foci (EPFs) are negative for CK-19 **A.** Photomicrograph illustrating CK-19 staining in bile ducts of control liver and proliferating oval cells 3 and 7 days after PH (CK-19, X10). Serial sections stained for hematoxylin-eosin (H&E) are depicted above (magnification X10). **B.** GSTP-positive EPFs developed 3 days after PH (left) are completely negative for CK-19 (magnification X4, left; X10, right). Few CK-19- positive oval cells are present. **C.** Photomicrograph showing that none of the GSTP+ EPFs occurring 7 days after PH are positive for CK-19 (magnification X1.25, left; X4, right). Several CK-19 positive oval cells are present.

Our gene expression analysis showed that, unlike other biliary markers up-regulated in EPFs (GSTP, GGT), CK-19 was poorly expressed in these lesions, suggesting this marker is not a built-in feature of precursor populations of HCC. Since our microarray was performed on pools of EPF, it was not possible to rule out the possibility that CK-19 was expressed in a small fraction of them. To investigate more in depth CK-19 expression in EPFs, we performed immunohistochemistry (IHC) analysis of CK-19 expression in oval cells and EPFs at 3 and 7 days after surgery. As seen in Figure 2A, while CK-19 positivity was detected in the vast majority of proliferating oval cells - which rapidly expand after PH in the presence of 2-AAF - IHC of more than 400 hepatocyte foci (performed on serial cryostat liver sections 3 and 7 days after PH) revealed that 100% of the GSTP-positive EFPs were completely negative for CK-19 (Figure 2B, 2C). The observation that no oval cells could be detected prior to PH and the concomitant presence of both this cell population and EPFs as early as 3 days after surgery, further suggest that EPFs do not derive from oval cells.

### CK-19 expression is acquired by a subset of nodules during their transition from a focal to a nodular stage

**Figure 3:**
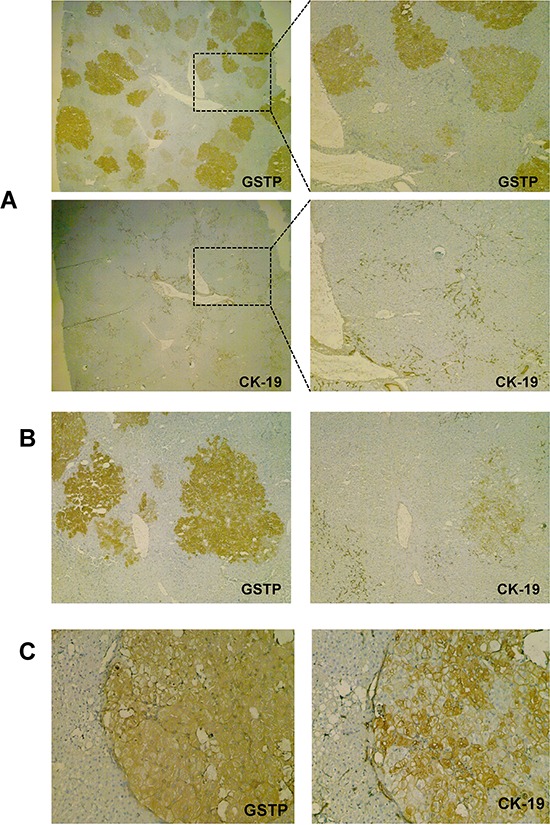
CK-19 positivity is acquired along with the progression from EPFs to a nodular stage **A.** Photomicrograph showing GSTP and CK-19 staining in preneoplastic lesions 2 weeks after PH (magnification X10). **B.** Photomicrograph showing occasional CK-19 staining in a nodule 2 weeks after PH (magnification X10), **C.** A GSTP-positive nodule showing a large number of cells positive for CK-19 at 7 weeks after PH (magnification X10).

The first evidence of CK-19 positivity was found 5 weeks after DENA (that is, 7 days after 2-AAF release); at that time, very few CK-19-positive preneoplastic nodules were detected in a context of otherwise negative lesions (19 CK-19+/250 GSTP+ nodules; 7.60%) (Figure 3A, 3B). In agreement with previous works [29, 30], at a later time point (10 weeks after carcinogen administration), about 25% of GSTP positive nodules showed positivity for CK-19 (Figure 3C). These nodules closely resemble human dysplastic nodules as they display hepatocytes with large eosinophilic cytoplasm and evident nucleoli [38]. These lesions often compress adjacent parenchyma and show distorted architecture. EPFs and CK-19+ nodules were characterized by a diffuse positivity to 5-bromo-2′ deoxyuridine (BrdU), which is indicative of very high proliferative activity (Supp. Figure 2A, 2B); however, unlike EPFs, they exhibited the presence of several cells positive for the cleaved form of caspase-3 (Cas-3) (Supp. Figure 3A, 3B, 3D), a typical feature of advanced HCC (Supp. Figure 3C, 3D). Measurement of Cas-3 positive cells resulted in an apoptotic index (AI) of 0.01/field in EPFs, 4/field in CK-19+ nodules and 15/field in HCC. On the contrary, CK-19-negative nodules, accounting for the vast majority of the lesions observed at this time point, resemble regenerative nodules in a cirrhotic-like background and did not exhibit major cytological and/or architectural dysplastic changes. In agreement with our previous studies [39], they also showed a much lower number of BrdU-positive hepatocytes (Supp. Figure 2B), and virtually no positivity for caspase-3 (apoptotic index was 0.4/field) (Supp. Figure 3B and 3D).

### CK-19-negative EPFs exhibit a gene expression profile similar to CK-19-positive nodules and HCCs

**Figure 4:**
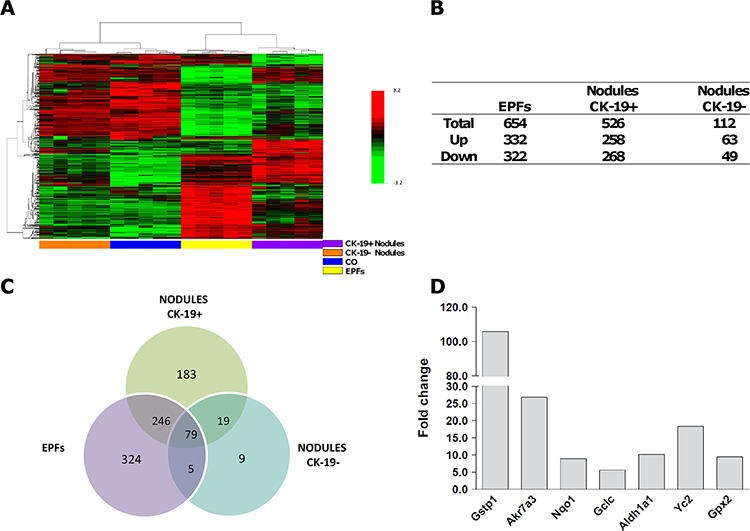
mRNA profile of EPFs, CK-19-positive and CK-19-negative nodules **A.** Hierarchical clustering of 1310 genes in normal liver (CO), EPFs and CK-19-positive and CK-19-negative nodules. Each row represents the expression profile of a gene. Columns represent a single sample for CK-19-positive and negative nodules and controls, and a pool of 10–15 EPFs. Controls and nodules are indicated by colored bars. Only mRNAs whose expression was dysregulated more than 2-fold were considered. Red and green colors represent higher or lower expression levels of the mRNA (median-centered), respectively. **B.** Number of differentially expressed mRNAs in EPFs, CK-19-positive and CK-19-negative nodules compared to age-matched controls, defined by *P* < 0.05 (BH-corrected). RNA was extracted from laser-microdissected early preneoplastic foci (EPFs) occurring 7 days after PH and CK-19-positive and CK-19-negative nodules generated 7 weeks after surgery. **C.** Venn diagrams illustrating overlapping subsets of significantly dysregulated genes in EPFs, CK-19-positive and CK-19-negative preneoplastic nodules. **D.** Microarray analysis of mRNA levels of Nrf 2-target genes in CK-19-negative EPFs developed 7 days after surgery. Values are expressed as fold increase compared to livers from age-matched controls.

It has been shown that CK-19-positive preneoplastic nodules exhibit a similar global expression profile to that of advanced HCCs, while CK-19-negative nodules co-cluster together with the control liver [30]. Therefore, we wished to investigate and compare the expression profile of early CK-19-negative EPFs with that of CK-19-positive and CK-19-negative preneoplastic nodules. Gene expression profiling was performed using the same early foci described above, and preneoplastic nodules CK-19-positive and CK-19-negative obtained 10 weeks after initiation. A total of 1,310 out of 21,791 genes included in the array were selected, as described in Supporting Material. Hierarchical cluster analysis stratified the lesions into two major clusters: 1) normal liver (CO) and preneoplastic CK-19-negative nodules; 2) CK-19-negative EPFs and CK-19-positive nodules (Figure 4A). As shown in the Venn diagram, EPFs and CK-19-positive nodules, unlike CK-19-negative nodules, exhibited a high number of modified genes compared to control livers (Figure 4B, 4C). Notably, while 324 genes were exclusively altered in EPFs and 183 in CK-19-positive preneoplastic nodules, 246 dysregulated genes were shared between EPFs and CK-19-positive nodules; only 5 genes were uniquely modified in EPFs and CK-19-negative nodules. These data suggest that EPFs are very likely to evolve directly to CK-19-positive preneoplastic lesions, while CK-19-negative nodules - whose expression profile is close to normal liver - are probably lesions transitioning towards a fully differentiated phenotype.

Among the differentially expressed genes, 16/20 of the most up-regulated (fold change versus controls >5) (Table 1) and 16/42 of the most down-regulated (fold change versus controls <5) genes in EPFs were the most dysregulated in CK-19-positive nodules as well (Table 2). Among the up-regulated genes in both EPFs and CK-19-positive nodules, target genes of the Nrf 2-Keap1 pathway were the most frequent (Figure 4D) indicating that activation of this pathway plays a critical role in the clonal expansion of initiated cells and their progression to malignancy. Remarkably, analysis of transcription factor-dependent pathways revealed that, when compared to EPFs and CK-19-positive nodules, CK-19-negative lesions showed a significant decrease of Nrf 2-pathway activation (51 and 48 dysregulated genes vs. 19 respectively) (Supp. Figure 4), suggesting that sustained activation of this pathway is linked to cancer development.

**Table 1:** Most up-regulated genes (>5)

Gene symbol	FC EPFs/ctr	Gene symbol	FC CK-19+/ctr
**Gstp1**	105.82	**Gstp1**	102.24
**Abcb1**	29.70	**Gstp2**	41.96
**Akr7a3**	26.79	**Akr1b8**	35.36
**Gstp2**	26.37	Defb1	34.28
**Akr1b8**	25.67	**Akr7a3**	29.13
**Yc2**	18.32	**Ca2**	26.91
**Slc25a4**	15.48	**Aldh1a1**	18.13
**Cyp2c40**	12.35	**Nqo1**	17.41
**Aldh1a1**	10.25	**Abcb1**	16.26
**Gpx2**	9.52	Pcp4	15.45
**Ca2**	9.27	**Gclc**	13.63
Smp2a	9.26	**Cyp2c40**	12.86
**Nqo1**	9.01	Nefl	12.82
**Abcc3**	6.91	**Abcc3**	12.79
A2m	5.96	**Yc2**	12.79
**Gclc**	5.64	**Ltb4dh**	12.76
**Ltb4dh**	5.41	**Gpx2**	12.36
Mt1a	5.33	Krt1–19	9.93
**Ephx1**	5.20	Maf	8.96
Atp6v1d	5.12	**Slc25a4**	8.15
		Ddit4l	7.62
		Gclm	6.83
		Cd24	6.79
		Tubb6	6.70
		S100a11	6.58
		Cryl1	6.28
		Slc17a3	6.15
		**Ephx1**	6.15
		Slc20a1	6.15
		Ctse	6.05
		Anxa2	6.00
		Bzrp	5.71
		Igfbp1	5.56
		Ugdh	5.45
		Ugt1a6	5.39
		Tacstd1	5.12

Genes in bold are up-regulated in EPFs and in CK-19+ nodules as well. Gene expression is reported as fold-change relative to age-matched controls.

**Table 2:** Most down-regulated genes (<−5)

Gene symbol	FC EPFs/ctr	Gene symbol	FC CK-19+/ctr
**G6pc**	−5.10	Cyp4a14	−4.91
Amacr	−5.13	Akr1c21	−5.66
Baat	−5.16	G0s2	−6.18
**Ust5r**	−5.22	Cyp8b1	−6.20
Cyp2b15	−5.31	**Ust5r**	−6.55
Pgcp	−5.46	**Ste2**	−6.60
Sez6	−5.51	**Cyp2c7**	−6.62
**Cyp2c7**	−5.57	**G6pc**	−6.95
Ndrg2	−5.60	Cyp2c37	−7.08
Dio1	−5.72	**Aox3**	−7.09
**Slc27a5**	−5.73	**Hao2**	−7.39
**Avpr1a**	−5.76	**Avpr1a**	−7.40
**Oat**	−5.84	Cyp3a11	−7.57
Slco1b2	−5.89	Cyp1a2	−7.59
Rnase4	−5.91	**Oat**	−7.99
Thrsp	−6.34	**Slc27a5**	−8.09
Hba-a1	−6.36	**Olr59**	−8.42
Gnmt	−6.54	**Fabp7**	−8.42
Hbb	−6.59	**Ca3**	−8.52
**Cyp3a13**	−6.84	Cyp3a3	−9.02
Pklr	−7.06	**Cyp3a13**	−9.96
Mup5	−7.10	Cdh17	−10.20
Scd1	−7.30	Cyp3a1	−11.57
Serpina3m	−7.46	**Dhrs7**	−14.86
Ang1	−7.48	**Cyp2c**	−15.48
Mug2	−7.67	**Obp3**	−41.11
**Olr59**	−7.77		
**Fabp7**	−7.87		
**Aox3**	−7.89		
Serpina4	−7.96		
Ces3	−8.77		
Sult1c1	−9.18		
Ppp1r3b	−9.52		
**Ste2**	−9.56		
Spin2b	−9.90		
**Hao2**	−10.87		
Apoa2	−11.14		
Mup4	−18.26		
**Ca3**	−20.20		
**Dhrs7**	−30.23		
**Obp3**	−62.76		
**Cyp2c**	−115.98		

Genes in bold are down-regulated in EPFs and in CK-19+ nodules as well. Gene expression is reported as fold-change relative to age-matched controls.

Notably, microarray analysis showed that the most dysregulated genes in EPFs matched with those previously identified in advanced HCC (Supp. Table 1 and Ref. 30). Quantitative RT-PCR validation performed on the three most up-regulated and the three most down-regulated genes in EPFs and HCCs confirmed microarray expression data (Supp. Figure 5). These results further support the notion that the major expression changes observed in HCC occur in the very first stages of the tumorigenic process.

**Figure 5:**
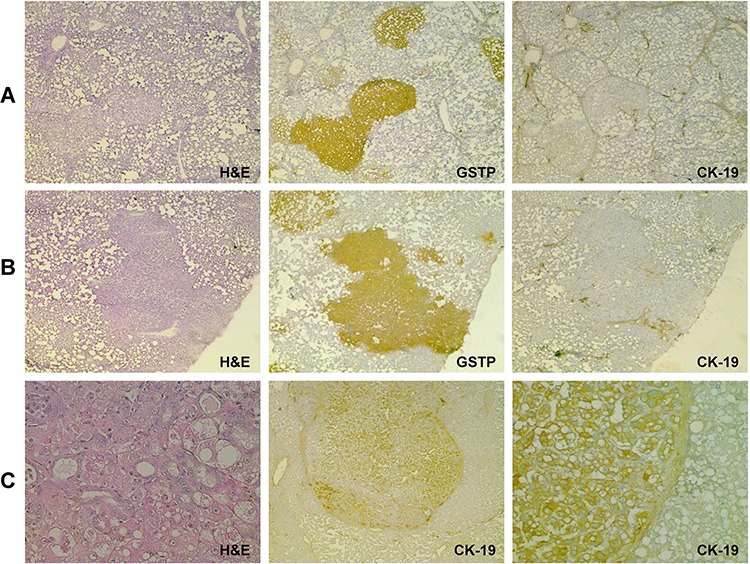
CK-19 positivity is a late event in the tumorigenic process induced by the CMD protocol of hepatocarcinogenesis Photomicrographs showing absence of CK-19 staining in: **A.** EPFs and **B.** preneoplastic nodules, occurring 10 week and 4 months after DENA treatment and strongly positive for GSTP. **C.** H&E (left) Photomicrograph of a typical HCC developed 13 months after initiation (H&E, X20, left) showing a large number of CK-19-positive cells (middle, X10); larger view of the same HCC (right, X20).

In conclusion, the results demonstrate that the expression profile of CK-19-negative EPFs is essentially similar to that of CK-19-positive nodules developed 6 weeks later, and that activation of the Nrf 2/Keap1 pathway might dictate the fate of EPFs (persistency and progression vs. remodeling). They thus suggest that CK-19-positive hepatocytes do not originate from liver progenitor cells, but are the result of a reprogramming of mature, although altered, hepatocytes.

### Expression of CK-19 is acquired late in the carcinogenic process also in the CMD protocol

To investigate whether the lack of CK-19 expression in early stages of HCC development is unique to the R-H model of hepatocarcinogenesis, we studied a different rat model, consisting of a choline-devoid methionine-deficient diet [33] (Supp. Figure 1B), and characterized by extensive fatty liver. IHC analysis showed that in this model early foci appearing 10 weeks after initiation, as well as preneoplastic lesions detected 4 months after DENA, were also completely negative for CK-19 (Figure 5A, 5B). However, a diffuse CK-19 positivity was detected in 8/8 HCCs arising after 13 months of CMD diet feeding (Figure 5C). These results further support the concept that CK-19-positive hepatocytes are the consequence of a reprogramming of mature, although altered, hepatocytes.

## DISCUSSION

In this work we addressed the question of the origin of HCC cells in the context of the rat R-H and CMD models of hepatocarcinogenesis. The R-H model is characterized by the proliferation of oval cells soon after PH in the presence of the cytostatic environment generated by 2-AAF [27]. An increased number of oval cells, positive for CK-19 and other biliary markers, was observed up to 3–4 weeks after surgery [34]. For this reason, it has been hypothesized that preneoplastic cells derive from proliferating oval cells [35, 36]. Indeed, in the R-H model, most of HCCs are CK-19-positive while the majority of preneoplastic lesions are negative for this marker and are believed to undergo spontaneous regression, further suggesting that CK-19-positive lesions are the precursors of HCCs [27].

Data obtained from this as well as other rodent HCC models raised considerable discussion about the involvement of hepatic progenitor cells (oval cells in rodents) in liver carcinogenesis. Indeed, while Sell et al. [35, 36] postulated that the sequence of hepatocyte foci to nodules of increasing size and then to HCC most likely originates from bipotential oval cells, Anilkumar et al. [31] demonstrated an independent development of the ductular oval cell response and the emergence and expansion of foci in the R-H model.

Trying to shed light on this relevant topic, we decided to further dissect the R-H model, analyzing lesions occurring before the appearance of preneoplastic nodules. We thus examined foci appearing 3 days after PH. These lesions, identified by GSTP staining, consist of about 15–20 cells on average, with features typical of hepatocytes (large round nuclei and evident basophilic cytoplasm); of the over 400 early preneoplastic foci examined, none displayed CK-19 positivity, not even in single cells. However, the surrounding liver presented many CK-19-positive cells with clear oval cell phenotype. Four days later, we observed an obvious enlargement of the foci, which still remained totally negative for CK-19. When we analyzed preneoplastic lesions (2 weeks after PH), while almost all the nodules were consistently negative, we detected for the first time a limited number of lesions exhibiting CK-19 positivity (19/250; 7.6%). Also in these nodules, CK-19-positive cells maintained a typical hepatocyte phenotype. Seven weeks after PH, the liver was almost completely occupied by large nodules, a quarter of which were positive for CK-19. It is important to underline that CK-19-positive nodules, on average, contained more than 25% CK-19-positive cells. This is hardly in agreement with the hypothesis that the CK-19-positive cells might represent HCC cancer stem cells. This idea is further enforced by the observation that most of HCCs appearing 10–14 months after DENA administration are characterized by CK-19 positivity and express a high number of hepatocytes positive for this marker.

**Figure 6:**
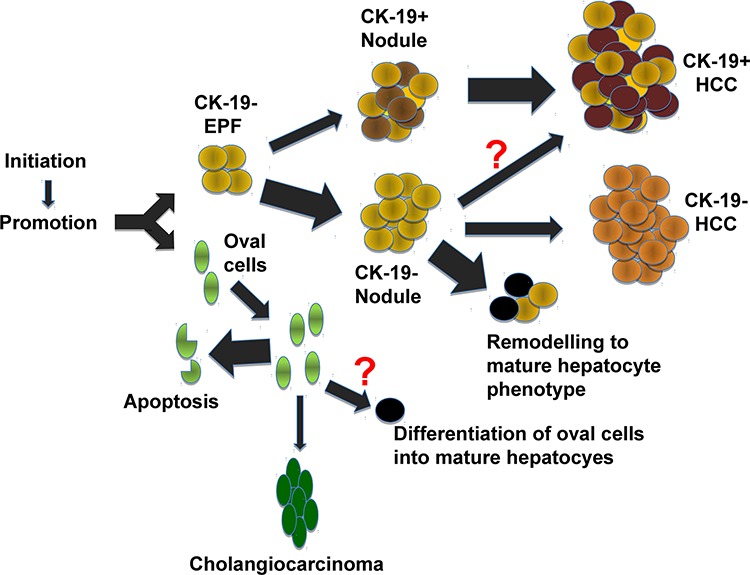
Representative scheme of the multistage tumorigenic process in the R-H model Exposure of rat liver to the R-H protocol gives rise to early preneoplastic foci (EPFs) and oval cells. While all EPFs are virtually negative for CK-19, two distinct types of nodules arise later on during the tumorigenic process: 1) CK-19-positive (a minority) and CK-19-negative (the majority) nodules. CK-19-positive nodules exhibit an expression profile very similar to that of EPFs and HCC, suggesting that they represent the precursor cell population of HCC. In contrast, most of the CK-19-negative nodules undergo remodeling and disappear. Few negative HCCs can be observed which probably originate from CK-19-negative nodules. Oval cells which actively divide during the first week post-surgery are no longer visible 3–4 weeks after PH. Occasionally, they can give rise to cholangiocarcinomas.

All these data suggest that the neoplastic lesions observed in the R-H model originate from CK-19-negative cells and that they acquire the expression of this marker only later, during the tumorigenic process (Figure 6). Similar data were also obtained in a different rat model consisting of feeding rats with a CMD diet, whereby lesions develop in a steato-necrotic environment caused by a choline devoid-methionine deficient diet. In fact, while HCCs are definitely CK-19-positive, no cells stained for this marker were observed at early or intermediate stages of the carcinogenic process.

Altogether these data suggest that not only normal liver cells but also neoplastic hepatic cells maintain a high level of plasticity, being able to acquire/lose markers considered restricted to defined cell types. Furthermore, we believe that this is very important from a translational point of view because it implies that markers/phenotypes observed in advanced cancers do not necessarily reflect the cell of origin of the tumor; they might instead be acquired/lost due to differences in the environment or to the distress conditions often present during tumorigenesis. This can be particularly true for molecules, like cytokeratins, which are involved in the maintenance of cyto-architecture and are particularly sensitive to mechanical stress [40].

Camargo and colleagues have recently shown that adult hepatocytes have the potential to give rise to cells that molecularly and functionally resemble liver progenitors or “oval” cells [41]. The observation that a large proportion of hepatocytes can undergo dedifferentiation suggests that most hepatocytes intrinsically harbor this developmental capacity. In our work we show that not only normal adult hepatocytes are inherently plastic and might dedifferentiate into a progenitor-like cell type, but that this property is maintained also by hepatocytes “primed” toward malignancy. These findings are of particular relevance also for human pathology, as CK-19-positive HCCs, endowed with worse prognosis, are believed to derive from oval cells or CK-19-positive progenitor cells. In this context, the analysis of the expression profiles performed in this work further reinforces the idea that the R-H neoplastic lesions do not originate from oval cells. As shown in Figure 1A, in fact, expression profiles of early foci and oval cells are clearly distinct, while early foci co-cluster with control hepatocytes. It would be interesting to perform similar analyses in human livers as well, in order to verify the origin of CK-19-positive tumors directly.

Another interesting point stemming from the present work is the evolutionary landscape of progressive lesions in the R-H model. Expression profiles show that EPFs co-cluster with CK-19-positive nodules, while CK-19-negative ones are more similar to normal liver. In this frame, we can hypothesize that, upon the carcinogenic stimulus, hepatocytes undergo a profound metabolic change that is required for further progression. Indeed, in other works we have shown that the gene expression profile of CK-19-positive nodules is very similar to that of advanced HCC [29, 30]. CK-19-negative nodules, instead, undergo remodeling, as testified by a progressive loss of neoplastic markers (such as GSTP, GGT), and reacquisition of an expression pattern typical of normal hepatocytes; these phenotypic changes precede nodule disappearance [42, 43]. Notably, a similar alternative fate of hepatic dysplastic nodules has been observed in humans. Indeed, as reported by Roncalli et al., only a minority of regenerative/dysplastic nodules undergo malignancy, while 40–60% stabilize, and a few definitely disappear during patients’ follow-up [44].

Although the mechanisms responsible for the alternative fate of preneoplastic nodules are unknown, it is interesting to note that many of the genes found up-regulated in both EPFs and CK-19-positive nodules are targets of Nrf 2, a transcriptional factor that upon nuclear translocation induces the expression of genes involved in cytoprotection and proliferation [45–47]. The finding that the Nrf 2-Keap1 pathway is activated in very early and small preneoplastic foci and that its activation persists only in those nodules that are considered precursors of HCC in the R-H model [30] makes Nrf 2 an interesting and promising therapeutic target in HCC therapy and suggests its potential prognostic use for the identification of progressive lesions in human HCC development. Notably, our very recent data obtained in the R-H model showed that activation of NRF2/KEAP1 pathway, due either to down-regulation of miRNA-200a, which targets KEAP1, or to extremely frequent missense mutations of NRF2, characterizes preneoplastic nodules and persists all throughout the process up to HCC development [48]. In the same study, we also showed that following injection of NRF2-silenced HCC cells into syngeneic rats, the tumorigenic capacity of a rat HCC cell line was completely abolished.

In conclusion, our present results obtained in two different rat models of hepatocarcinogenesis demonstrate that CK-19 expression is not intrinsic to preneoplastic cells, but is acquired later in the tumorigenic process, and does not necessarily predict the cell origin of HCC. This work thus demonstrates that not only normal adult hepatocytes but also preneoplastic ones are inherently plastic and can acquire markers of more indifferentiated cells.

## MATERIALS AND METHODS

### Animals and treatment

#### R-H protocol

Male Fischer rats were obtained from Charles River, Milano, Italy. Guidelines for Care and Use of Laboratory Animals were followed during the investigation. All animal procedures were approved by the Ethical Commission of the University of Cagliari and the Italian Ministry of Health. Animals were treated with a single dose of diethylnitrosamine (DENA, 150 mg/kg, Sigma-Aldrich, Milano, Italy) and, two weeks later, were subjected to the R-H protocol, consisting of a 2-week diet supplemented with 0.02% 2-acetylaminofluorene (2-AAF) and a two/thirds partial hepatectomy (PH) [27]. Rats were sacrificed 3 or 7 days after PH or switched to basal diet all throughout the experiment and sacrificed 5 and 10 weeks after DENA administration (Supp. Figure 1A).

#### CMD protocol

Four week-old male F-344 rats (90–100grams) were given DENA (150 mg/kg) and 2 weeks later fed a choline-methionine deficient (CMD) diet [33]. Rats were sacrificed 10 weeks and 4 and 13 months after DENA administration (Supp. Figure 1B).

### Immunohistochemistry

Immediately after sacrifice, liver sections were fixed in 10% formalin or snap-frozen in liquid nitrogen and processed for hematoxylin-eosin, cresyl violet, GSTP, CK-19, and BrdU immunohistochemistry, as described [30]. We defined CK-19-positive nodules as all those lesions exhibiting a CK-19-positive area of at least 5% of the total area of the preneoplastic lesion (the criteria commonly used by pathologists). The average area occupied by CK-19-positive hepatocytes was higher than 25% of the total area of the preneoplastic nodules. We considered as CK-19-negative all those lesions which did not exhibit any CK-19-positive cells within the preneoplastic lesion, either EPFs or nodules. Immunostaining for the cleaved form of caspase 3 was performed to detect cell death, according to Eckle et al [49]. The apoptotic index is expressed as number of cleaved-caspase-3 (Cas-3) positive cells/field (20x). 30 EPFs, 12 CK-19+ nodules and 36 CK-19- nodules were scored.

#### Laser-capture Micro-dissection (LMD)

we microdissected 60 foci (1 week after PH), 10 nodules (10 weeks after initiation with DENA), and random areas from the liver of rats exposed to the R-H protocol. Oval cells, identified on morphological criteria after staining with cresyl violet and H&E, and by immunohistochemistry (CK-19, GSTP, GGT), were then microdissected from cresyl violet stained sections 1 week after PH (for details see Supp. Material).

#### mRNA expression profiling

RNA was extracted and purified from oval cells or preneoplastic foci and nodules after laser microdissection from the liver of four to five animals. For the gene expression profile, 150 ng of RNA were amplified (Illumina TotalPrep RNA Amplification Kit), labeled and hybridized on Illumina microarray (RatRef-12 V1 BeadChips, Illumina Inc., San Diego, CA, USA), including 21.791 genes (for further details and data analysis see Supporting Material). mRNAs validation was performed using specific TaqMan assays (Applied Biosystems). To identify the differentially expressed genes in each type of group towards its age-matched control we applied the Random-Variance Model and Multivariate Permutation Test. Raw microarray data have been deposited in the GEO database (http://www.ncbi.nlm.nih.gov/geo/query/acc.cgi?acc=GSE70322) with Accession Number GSE70322.

### qRT-PCR analysis

RNA was retro-transcribed using the High Capacity cDNA Reverse Transcription Kit (Life Technologies). Analysis of Epcam, CK-19, Nqo1, Akr1b8, Defb1, Gstp1, Ca3, Dhrs7 and Hao2 was performed using specific TaqMan probes (Life Technologies) and GAPDH as the endogenous control.

#### Statistical analysis

One-way analysis of variance (ANOVA) and Student's *t*-test were used to analyze the data (Instat; GraphPad, San Diego, CA, USA). The results of observations are presented as the means ± SE. A value of *P* < 0.05 was regarded as a significant difference between groups.

## SUPPLEMENTARY DATA TABLE AND FIGURES



## Abbreviations

CK-19: cytokeratin-19
HCC: hepatocellular carcinoma
HPC: hepatic progenitor cell
R-H model: Resistant-Hepatocyte model
CMD: choline-methionine deficient diet
EPF: early preneoplastic foci
qRT-PCR: quantitative reverse transcriptase polymerase chain reaction
DENA: diethylnitrosamine
AAF: 2-acetylaminofluorene
PH: partial hepatectomy
GSTP: placental glutathione S-transferase
Epcam: epithelial cell adesion molecule
NQO1: NAD(P)H dehydrogenase, quinone 1
GAPDH: glyceraldehyde 3-phosphate dehydrogenase
BrdU: 5-Bromo-2′-deoxyuridine
Cas3: cleaved Caspase 3
GGT: gamma-glutamyil transpeptidase
IHC: immunohistochemistry
GCLC: glutamate-cysteine ligase
GSTA4: glutathione S-transferase A4
IPA: ingenuity pathway analysis
KEAP1: kelch-like ECH-associated protein 1
NRF2: nuclear factor (erythroid-derived 2)-like 2
AKR1b8: aldo-keto reductase 1, member b8
DEFB1: Defensin b1
Ca3: Carbonic anhydrase 3
DHR7: dehydrogenase/reductase member 7.
