# Supplementary material for: Cytokeratin-19 positivity is acquired along cancer progression and does not predict cell origin in rat hepatocarcinogenesis
Source: Oncotarget. 2015 Oct 3;6(36):38749–63. doi: 10.18632/oncotarget.5501 (PMC4770734; doi:10.18632/oncotarget.5501)
Supplement: Supplementary file 1 [file oncotarget-06-38749-s001.pdf]

# SUPPLEMENTARY DATA

## Laser microdissection

We microdissected 60 early preneoplastic foci (EPFs) (1 week after PH), 10 nodules (10 weeks after initiation with DENA) and random areas of normal liver. Oval cells were also microdissected 1 week after PH. Sixteen-µm-thick serial frozen sections of rat livers were attached to 2-µm RNase free PEN-membrane slides (Leica, Wetzlar, Germany). Prior to microdissection (Leica, LMD6000), slides were stained with an H&E modified protocol [1]. RNA was extracted from micro-dissected preneoplastic lesions and oval cells using either the PicoPure RNA Isolation Kit (Arcturus, Mountain View, CA, USA), or MirVana (Life Technology, Milano, Italy), as previously reported (1).

## Microarray data analysis

The intensity files were loaded into the Illumina BeadStudio 3.0.19.0 software (Illumina Inc, San Diego, CA, USA) and BRB Array Tools (Version 4.4.0) for quality control and gene expression analysis. First, the quantile normalization algorithm was applied on the dataset. Only genes whose expression differed by at least 1.5 fold from the median in at least 20% of the arrays and characterized by a 50th percentile of intensities greater than 300 were retained. The FDR-adjusted *p*-values were calculated using the Benjamini-Hochberg procedure (2). According to these criteria, 1.570 expressed transcripts out of 21.791 showed reproducible up- or down-regulation in the first experiment (oval cells and early preneoplastic foci (EPFs) appearing 7 days after PH), while 1.310 expressed transcripts out of 21.791 showed reproducible up- or down-regulation between EPFs, CK-19-positive and CK-19-negative preneoplastic nodules. F-test (with random variance model) and multivariate permutation test were used to identify genes differentially expressed. We considered genes statistically significant if their *P value was less than* 0.01 with 80% confidence level of false discovery rate assessment and 0.1 maximum allowed proportion of false-positive genes. Following this analysis, 1545 genes showed reproducible up- or down-regulation in oval cells and EPFs, while 1295 genes showed reproducible up- or down-regulation in EPFs, CK-19-positive and CK-19-negative nodules. The *P* value ranged from $< 1e\text{-}07$ to 0.0081 for the comparison described in Figure 1C and from $< 1e\text{-}07$ to $< 0.056$ for the comparison described in Figure 4C.

## Functional analysis by means of the ingenuity IPA software

Rat standard gene symbols (RGD ids) were submitted to the Ingenuity IPA analysis pipeline. Analysis of “Transcription Factors regulators” was based on the number of genes significantly dysregulated (fold difference cut off ± 2.0) with corresponding biological functions and was used to identify transcription factors whose targets are enriched in our datasets.

# REFERENCES


1. Perra A, Kowalik MA, Ghiso E, Ledda-Columbano GM, Di Tommaso L, Angioni MA, Raschioni C, Testore E, Roncalli M, Giordano S, Columbano A. YAP activation is an early event and a potential therapeutic target in liver cancer development. J Hepatol. 2014; 61:1088–1096.
2. Benjamini Y, Hochberg Y. Controlling the false discovery rate: a practical and powerful approach to multiple testing. Journal of the Royal Statistical Society Series B (Methodological). 1995; 57:289–300.

## Supplementary Table S1: Most dysregulated genes in HCC and EPFs

| Most up-regulated genes in rat HCC |
|---|
| **Akr1b8** |
| **Defb1** |
| **Gstp1** |
| **Gstp2** |
| **Gpx2** |
| **Ca2** |
| **Yc2** |
| **Nqo1** |
| Ddit4l |
| **Akr7a3** |
| Krt1–19 |
| Pcp4 |
| **Most down-regulated genes in rat HCC** |
| Cyp2c |
| **Obp3** |
| **Ca3** |
| **Dhrs7** |
| **Hao2** |
| **Cdh17** |
| **Olr59** |
| **Avpr1a** |
| **Sult1c1** |
| **Aox3** |
| **Cyp3a3** |
| **Ust5r** |
| **Cyp2c37** |
| Akr1c18 |
| **Gnmt** |

Genes in bold are dysregulated for >5 fold in EPFs as well

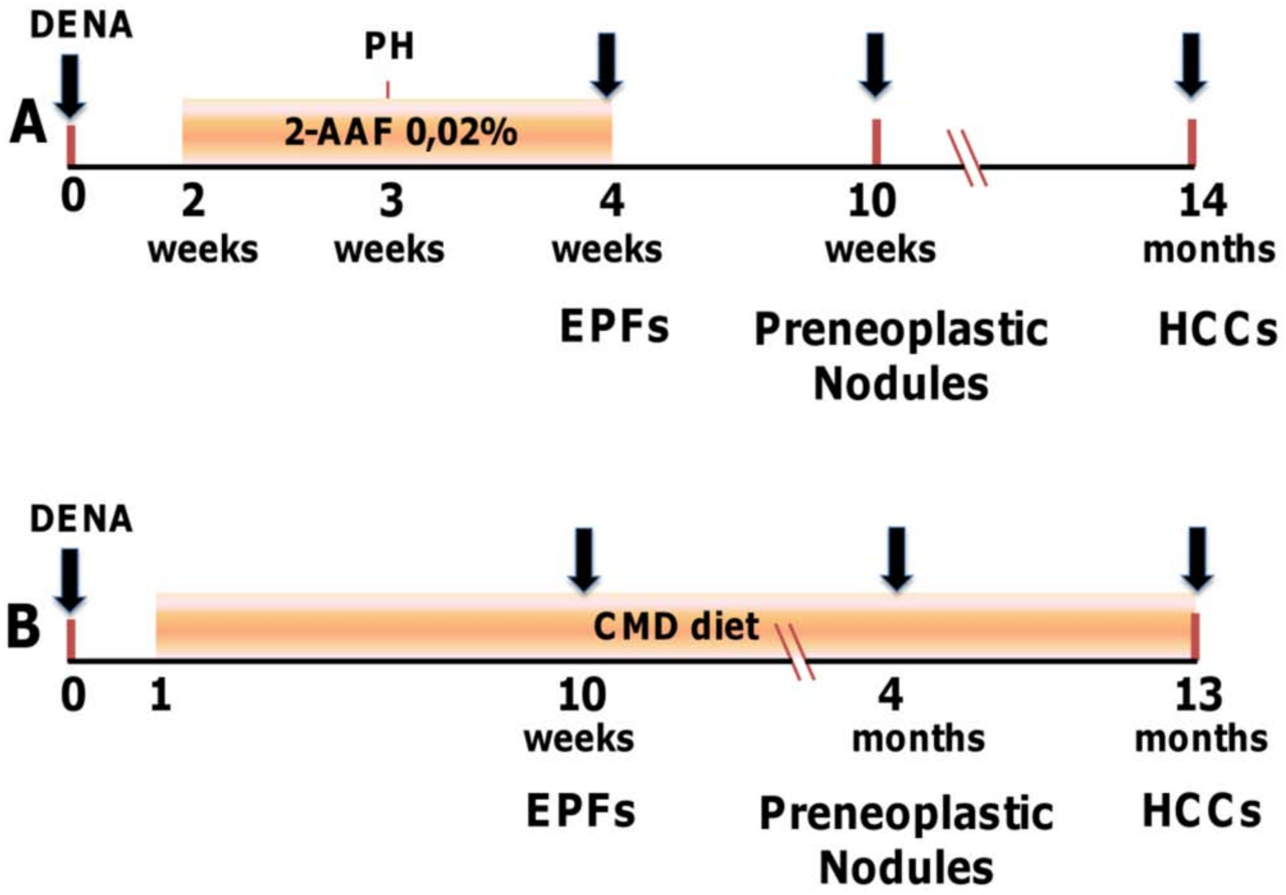


**Supplementary Figure S1: Schematic representation of the experimental protocols.** **A.** R-H model; **B.** CMD model.

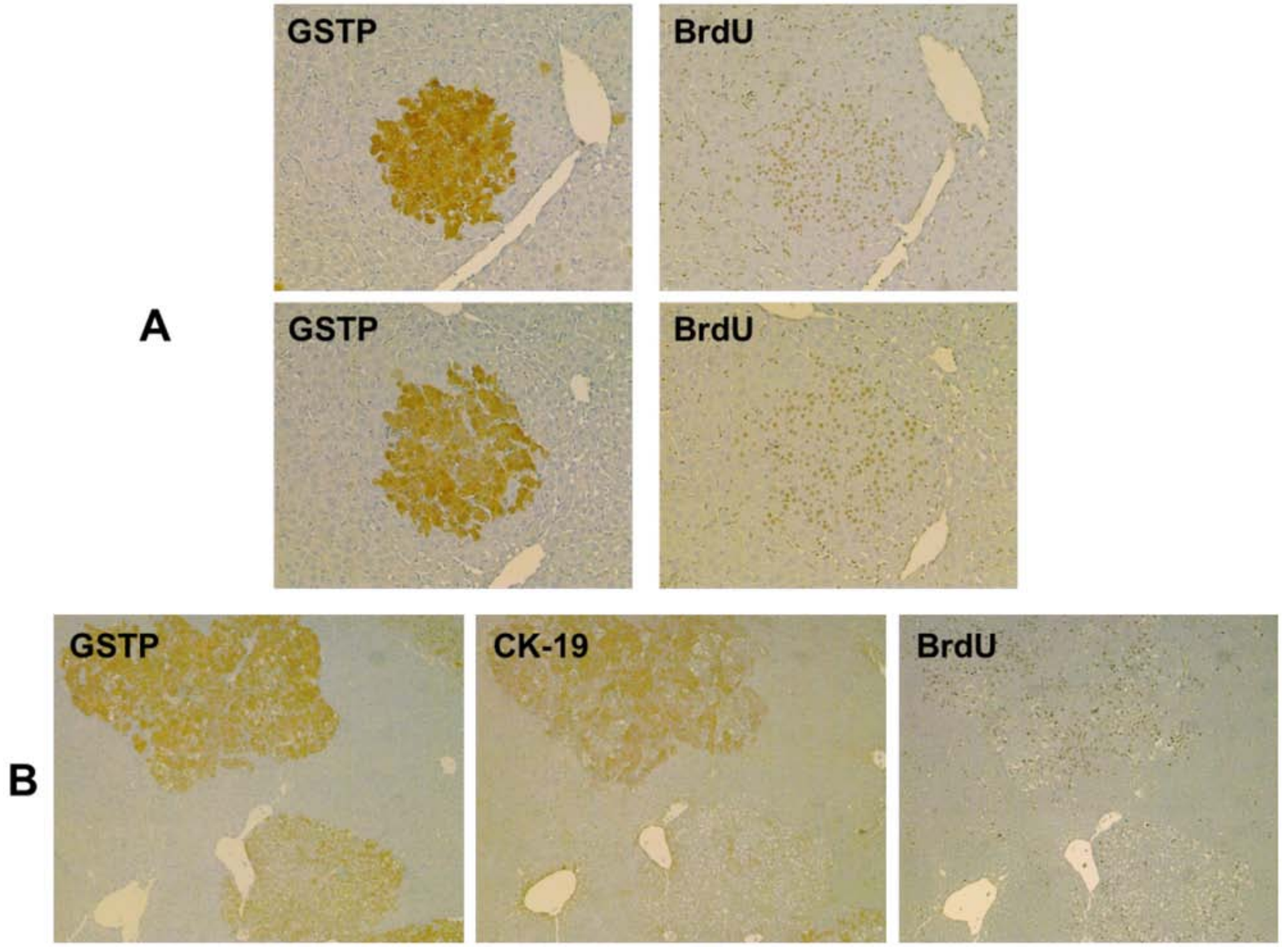


**Supplementary Figure S2: BrdU incorporation in EPFs and nodules. A.** Serial sections from rat livers showing two distinct GSTP+ EPFs (left) generated 4 weeks after treatment with DENA. Diffuse BrdU incorporation is present in both the EPFs (right) (x10); **B.** serial sections from rat liver showing GSTP+/CK-19+ and GSTP+/CK-19- nodules generated 10 weeks after treatment with DENA. Extensive BrdU staining is present in the CK-19+, but not in the CK-19- nodule (x4).

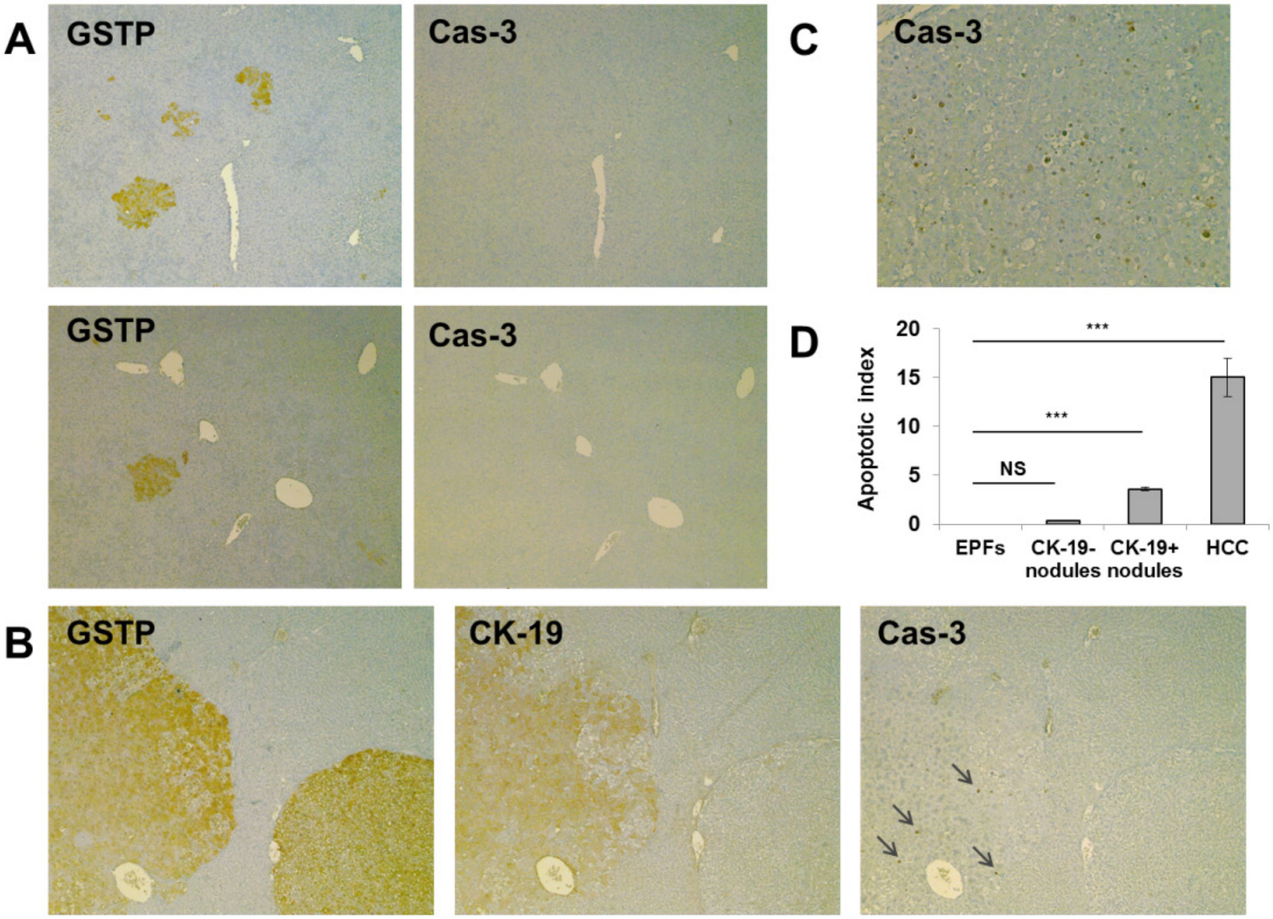


**Supplementary Figure S3: Cleaved-Caspase-3 immunostaining in EPFs and nodules. A.** Serial sections from rat livers showing no Cas-3 staining in GSTP+ EPFs generated 4 weeks after treatment with DENA. (x4); **B.** serial sections from rat liver showing GSTP+/CK-19+ and GSTP+/CK-19- nodules generated 10 weeks after treatment with DENA. Several Cas-3 positive cells (arrows) are present in the CK-19+, but not in the CK-19- nodule (x4); **C.** Cas-3 positive cells in a HCC developed 14 months after treatment with DENA (x10); **D.** Apoptotic index (AI). AI is expressed as number of Cas-3-positive cells/field. From 10 to 30 fields were examined. Results are expressed as Mean ± SE. ***$P < 0.001$.

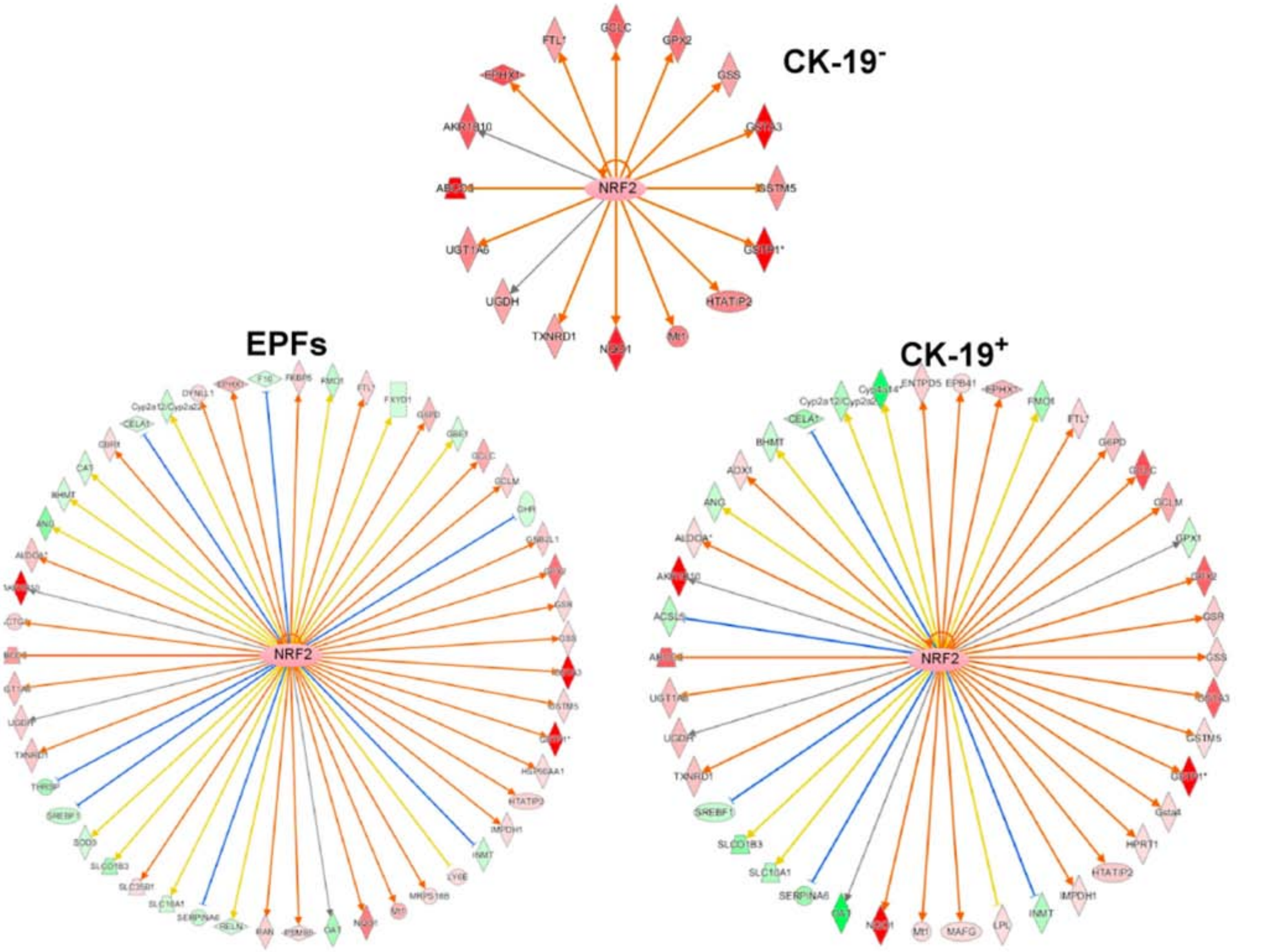


**Supplementary Figure S4: Network visualization of the transcription factor Nrf2 pathway.** Data and visualization were obtained using IPA. Red: up-regulated genes; green: downregulated genes.

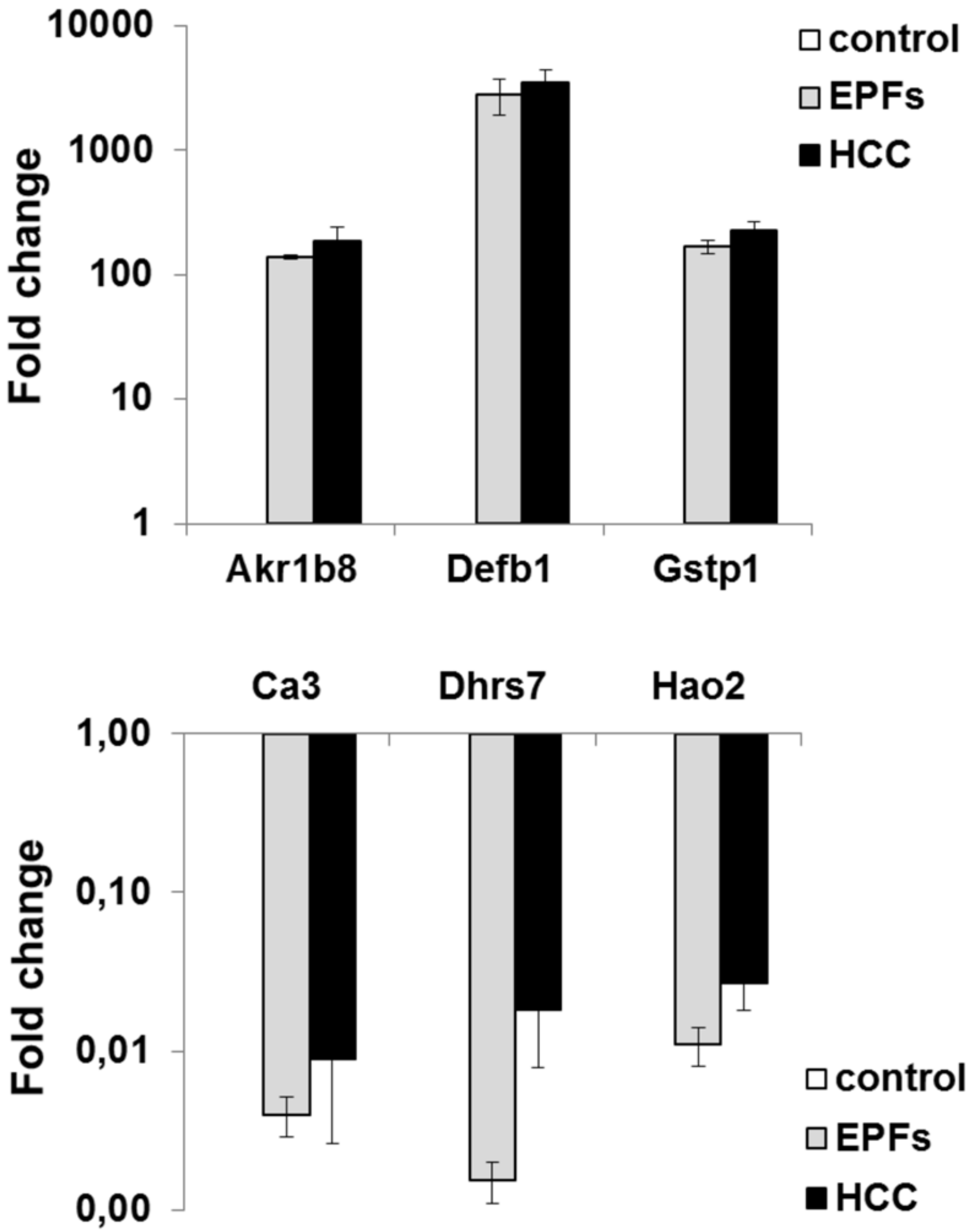


**Supplementary Figure S5: QRT-PCR validation analysis of the most up- and down-regulated genes commonly dysregulated in EPFs and HCC.** The values are reported as log fold change of HCC and EPFs vs.control liver. Mean ± SE of at least 5 samples/group. All values were significantly different from control for at least $P < 0.0001$.
